# *N*,*N*′-Bis(2-methyl­phen­yl)-*N*′′-(2,2,2-trichloro­acet­yl)phospho­ric triamide

**DOI:** 10.1107/S1600536811039511

**Published:** 2011-09-30

**Authors:** Mehrdad Pourayoubi, Mojtaba Keikha, Masood Parvez

**Affiliations:** a Department of Chemistry, Ferdowsi University of Mashhad, Mashhad 91779, Iran; b Department of Chemistry, The University of Calgary, 2500 University Drive NW, Calgary, Alberta, Canada T2N 1N4

## Abstract

In the title compound, C_16_H_17_Cl_3_N_3_O_2_P, the P—N bonds in the P(O)[NH(2—CH_3_)C_6_H_4_]_2_ unit [1.623 (4) and 1.637 (3) Å] are shorter than the P—N bond in the C(O)NHP(O) fragment [1.704 (3) Å]. The phosphoryl and carbonyl groups are *anti* with respect to each other and the P atom has a distorted tetra­hedral configuration. In the crystal, adjacent mol­ecules are linked *via* N—H⋯O(P) and N—H⋯O(C) hydrogen bonds into an extended chain parallel to [101].

## Related literature

For background to compounds having a C(O)NHP(O) skeleton, see: Toghraee *et al.* (2011 ▶); Pourayoubi, Tarahhomi *et al.* (2011 ▶). For bond lengths and angles in a related structure, see: Pourayoubi, Fadaei & Parvez (2011 ▶).
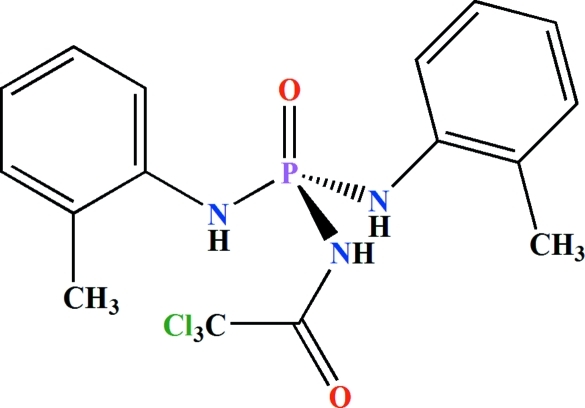


## Experimental

### 

#### Crystal data

C_16_H_17_Cl_3_N_3_O_2_P
*M* *_r_* = 420.65
Monoclinic, 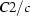

*a* = 14.2030 (5) Å
*b* = 16.1935 (6) Å
*c* = 16.9107 (6) Å
β = 102.3720 (19)°
*V* = 3799.1 (2) Å^3^
*Z* = 8
Mo *K*α radiation
μ = 0.58 mm^−1^
*T* = 173 K
0.10 × 0.09 × 0.08 mm

#### Data collection

Nonius KappaCCD diffractometer with APEXII CCD
Absorption correction: multi-scan (*SORTAV*; Blessing, 1997 ▶) *T*_min_ = 0.944, *T*_max_ = 0.955
8110 measured reflections
4296 independent reflections
3031 reflections with *I* > 2σ(*I*)
*R*_int_ = 0.055

#### Refinement

*R*[*F* ^2^ > 2σ(*F* ^2^)] = 0.071
*wR*(*F* ^2^) = 0.141
*S* = 1.11
4296 reflections
228 parameters
H-atom parameters constrained
Δρ_max_ = 0.48 e Å^−3^
Δρ_min_ = −0.38 e Å^−3^

### 

Data collection: *COLLECT* (Hooft, 1998 ▶); cell refinement: *DENZO* (Otwinowski & Minor, 1997 ▶); data reduction: *SCALEPACK* (Otwinowski & Minor, 1997 ▶); program(s) used to solve structure: *SIR92* (Altomare *et al.*, 1993 ▶); program(s) used to refine structure: *SHELXL97* (Sheldrick, 2008 ▶); molecular graphics: *Mercury* (Macrae *et al.*, 2008 ▶); software used to prepare material for publication: *SHELXL97* and *enCIFer* (Allen *et al.*, 2004 ▶).

## Supplementary Material

Crystal structure: contains datablock(s) global, I. DOI: 10.1107/S1600536811039511/jj2101sup1.cif

Structure factors: contains datablock(s) I. DOI: 10.1107/S1600536811039511/jj2101Isup2.hkl

Additional supplementary materials:  crystallographic information; 3D view; checkCIF report

## Figures and Tables

**Table 1:** Hydrogen-bond geometry (Å, °)

*D*—H⋯*A*	*D*—H	H⋯*A*	*D*⋯*A*	*D*—H⋯*A*
N1—H1⋯O2^i^	0.88	1.90	2.768 (4)	170
N2—H2⋯O1^ii^	0.88	2.11	2.957 (4)	162
N3—H3⋯O1^ii^	0.88	2.39	3.149 (4)	144

Symmetry codes: (i) 
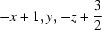
; (ii) 
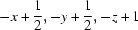
.

**Fig. 1.**
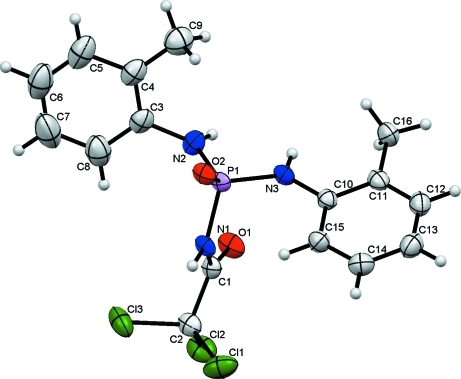
The molecular structure of the title compound with ellipsoids shown at the 50% probability level. [Colour key: P atom is violet, O atoms are red, N atoms are blue, Cl atoms are green and C and H atoms are light grey.]

**Fig. 2.**
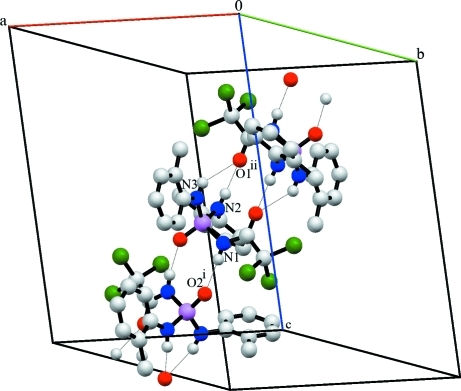
Part of the crystal structure of the title compound with N—H···O hydrogen bonds shown as dotted lines (the hydrogen atoms of the C—H units are omitted for clarity). [Symmetry codes: (i)-x+1, y, -z+1.5; (ii) -x+0.5, -y+0.5, -z+1]

## supplementary crystallographic information

### Comment

The structure determination of the title compound, P(O)[NHC(O)CCl_3_][NHC_6_H_4_(2-CH_3_)]_2_ (Fig. 1), was performed in continuing of works on the synthesis and structural investigation of new phosphoramidate compounds having a C(O)NHP(O) skeleton (Toghraee *et al.*, 2011; Pourayoubi, Tarahhomi *et al.*, 2011).

The phosphoryl and the carbonyl groups adopt the *anti* positions with respect to each other. The P atom has a distorted tetrahedral conformation. The bond angles around the P atom are in the range of 102.06 (17)° to 117.28 (17)°. As expected, the P1—N2 (1.623 (4) Å) and P1—N3 (1.637 (3) Å) bonds are shorter than the P1—N1 (1.704 (3) Å) bond. The P═O and C═O bond lengths and the P—N—C bond angles are standard for this category of compounds (Pourayoubi, Fadaei & Parvez, 2011).

In the crystal structure, adjacent molecules are linked *via* N_C(O)NHP(O)_—H···O(P) and N—H···O(C) hydrogen bonds, into an extended chain parallel to [101], Table 1 and Fig. 2.

### Experimental

CCl_3_C(O)NHP(O)Cl_2_ was synthesized from the reaction between phosphorus pentachloride (16.7 mmol) and 2,2,2-trichloroacetamide (16.7 mmol) in dry CCl_4_ at 358 K (3 h) and then the treatment of formic acid 85% (16.7 mmol) at ice bath temperature. To a solution of CCl_3_C(O)NHP(O)Cl_2_ (1.79 mmol) in CHCl_3_, a solution of *o*-toluidine (7.16 mmol) in CHCl_3_ was added dropwise at 273 K. After 4 h of stirring, the solvent was evaporated at room temperature. The solid was washed with distilled water. Single crystals were obtained from a mixture of CH_3_OH/CH_3_CN after slow evaporation at room temperature.

### Refinement

H-atoms were included in geometrically idealized positions with N—H = 0.98 Å and C—H = 0.95 and 0.98Å for aryl and methyl type H-atoms, respectively, and were included in the refinement with *U*_iso_(H) = 1.2*U*_eq_(C/N).

### Crystal data

**Table:**

C_16_H_17_Cl_3_N_3_O_2_P	*F*(000) = 1728
*M**_r_* = 420.65	*D*_x_ = 1.471 Mg m^−^^3^
Monoclinic, *C*2/*c*	Mo *K*α radiation, λ = 0.71073 Å
Hall symbol: -C 2yc	Cell parameters from 4137 reflections
*a* = 14.2030 (5) Å	θ = 2.5–27.5°
*b* = 16.1935 (6) Å	µ = 0.58 mm^−^^1^
*c* = 16.9107 (6) Å	*T* = 173 K
β = 102.3720 (19)°	Prism, colorless
*V* = 3799.1 (2) Å^3^	0.10 × 0.09 × 0.08 mm
*Z* = 8	

### Data collection

**Table:**

Nonius KappaCCD diffractometer with APEXII CCD	4296 independent reflections
Radiation source: fine-focus sealed tube	3031 reflections with *I* > 2σ(*I*)
graphite	*R*_int_ = 0.055
ω and φ scans	θ_max_ = 27.5°, θ_min_ = 2.5°
Absorption correction: multi-scan (*SORTAV*; Blessing, 1997)	*h* = −18→18
*T*_min_ = 0.944, *T*_max_ = 0.955	*k* = −20→21
8110 measured reflections	*l* = −21→21

### Refinement

**Table:**

Refinement on *F*^2^	Primary atom site location: structure-invariant direct methods
Least-squares matrix: full	Secondary atom site location: difference Fourier map
*R*[*F*^2^ > 2σ(*F*^2^)] = 0.071	Hydrogen site location: inferred from neighbouring sites
*wR*(*F*^2^) = 0.141	H-atom parameters constrained
*S* = 1.11	*w* = 1/[σ^2^(*F*_o_^2^) + (0.*P*)^2^ + 26.070*P*] where *P* = (*F*_o_^2^ + 2*F*_c_^2^)/3
4296 reflections	(Δ/σ)_max_ < 0.001
228 parameters	Δρ_max_ = 0.48 e Å^−^^3^
0 restraints	Δρ_min_ = −0.38 e Å^−^^3^

### Special details

**Table:**

Geometry. All e.s.d.'s (except the e.s.d. in the dihedral angle between two l.s. planes) are estimated using the full covariance matrix. The cell e.s.d.'s are taken into account individually in the estimation of e.s.d.'s in distances, angles and torsion angles; correlations between e.s.d.'s in cell parameters are only used when they are defined by crystal symmetry. An approximate (isotropic) treatment of cell e.s.d.'s is used for estimating e.s.d.'s involving l.s. planes.
Refinement. Refinement of *F*^2^ against ALL reflections. The weighted *R*-factor *wR* and goodness of fit *S* are based on *F*^2^, conventional *R*-factors *R* are based on *F*, with *F* set to zero for negative *F*^2^. The threshold expression of *F*^2^ > σ(*F*^2^) is used only for calculating *R*-factors(gt) *etc*. and is not relevant to the choice of reflections for refinement. *R*-factors based on *F*^2^ are statistically about twice as large as those based on *F*, and *R*- factors based on ALL data will be even larger.

### Fractional atomic coordinates and isotropic or equivalent isotropic displacement parameters (Å^2^)

**Table:**

	*x*	*y*	*z*	*U*_iso_*/*U*_eq_	
Cl1	0.23160 (9)	0.15298 (9)	0.78649 (8)	0.0533 (4)	
Cl2	0.09485 (7)	0.26270 (8)	0.68962 (7)	0.0474 (3)	
Cl3	0.27140 (8)	0.32851 (9)	0.78607 (8)	0.0513 (3)	
P1	0.44011 (7)	0.21369 (7)	0.61189 (6)	0.0253 (2)	
O1	0.2270 (2)	0.2435 (2)	0.58675 (16)	0.0383 (7)	
O2	0.53687 (18)	0.20089 (18)	0.66214 (15)	0.0312 (6)	
N1	0.3656 (2)	0.2181 (2)	0.67847 (18)	0.0264 (7)	
H1	0.3904	0.2094	0.7301	0.032*	
N2	0.4268 (2)	0.2961 (2)	0.5559 (2)	0.0350 (8)	
H2	0.3841	0.2950	0.5098	0.042*	
N3	0.3973 (2)	0.1417 (2)	0.54605 (19)	0.0288 (7)	
H3	0.3750	0.1581	0.4959	0.035*	
C1	0.2710 (3)	0.2342 (2)	0.6560 (2)	0.0274 (8)	
C2	0.2186 (3)	0.2441 (3)	0.7272 (2)	0.0349 (10)	
C3	0.4812 (3)	0.3707 (3)	0.5776 (3)	0.0379 (10)	
C4	0.5651 (3)	0.3852 (3)	0.5521 (3)	0.0430 (11)	
C5	0.6135 (4)	0.4609 (3)	0.5734 (3)	0.0556 (15)	
H5	0.6720	0.4722	0.5570	0.067*	
C6	0.5758 (5)	0.5179 (4)	0.6178 (4)	0.0661 (17)	
H6	0.6082	0.5691	0.6306	0.079*	
C7	0.4923 (6)	0.5032 (4)	0.6446 (4)	0.0733 (19)	
H7	0.4682	0.5432	0.6762	0.088*	
C8	0.4446 (4)	0.4301 (3)	0.6250 (3)	0.0565 (14)	
H8	0.3869	0.4191	0.6431	0.068*	
C9	0.6031 (4)	0.3245 (3)	0.5022 (3)	0.0573 (14)	
H9A	0.6592	0.3478	0.4850	0.069*	
H9B	0.5531	0.3108	0.4544	0.069*	
H9C	0.6223	0.2743	0.5340	0.069*	
C10	0.3923 (3)	0.0558 (3)	0.5609 (2)	0.0284 (9)	
C11	0.3738 (3)	0.0013 (3)	0.4949 (3)	0.0319 (9)	
C12	0.3716 (3)	−0.0828 (3)	0.5109 (3)	0.0384 (10)	
H12	0.3599	−0.1207	0.4670	0.046*	
C13	0.3860 (3)	−0.1126 (3)	0.5889 (3)	0.0426 (11)	
H13	0.3840	−0.1703	0.5983	0.051*	
C14	0.4031 (3)	−0.0585 (3)	0.6528 (3)	0.0391 (10)	
H14	0.4121	−0.0790	0.7065	0.047*	
C15	0.4074 (3)	0.0250 (3)	0.6399 (3)	0.0345 (10)	
H15	0.4206	0.0619	0.6846	0.041*	
C16	0.3589 (3)	0.0325 (3)	0.4097 (3)	0.0421 (11)	
H16A	0.3531	−0.0143	0.3723	0.051*	
H16B	0.4141	0.0667	0.4040	0.051*	
H16C	0.2999	0.0657	0.3970	0.051*	

### Atomic displacement parameters (Å^2^)

**Table:**

	*U*^11^	*U*^22^	*U*^33^	*U*^12^	*U*^13^	*U*^23^
Cl1	0.0420 (6)	0.0706 (9)	0.0466 (7)	−0.0022 (6)	0.0080 (5)	0.0227 (6)
Cl2	0.0251 (5)	0.0693 (9)	0.0452 (7)	0.0064 (5)	0.0018 (5)	−0.0046 (6)
Cl3	0.0384 (6)	0.0659 (8)	0.0473 (7)	0.0042 (6)	0.0041 (5)	−0.0278 (6)
P1	0.0242 (5)	0.0315 (6)	0.0181 (5)	−0.0012 (4)	0.0003 (4)	0.0003 (4)
O1	0.0291 (15)	0.057 (2)	0.0239 (15)	0.0055 (14)	−0.0050 (12)	0.0003 (14)
O2	0.0252 (14)	0.0454 (18)	0.0206 (14)	0.0009 (13)	−0.0009 (11)	−0.0016 (13)
N1	0.0251 (16)	0.0362 (19)	0.0149 (15)	0.0026 (14)	−0.0026 (12)	−0.0019 (14)
N2	0.0348 (19)	0.038 (2)	0.0263 (18)	−0.0065 (16)	−0.0060 (15)	0.0049 (16)
N3	0.0327 (18)	0.0355 (19)	0.0160 (16)	0.0003 (15)	0.0004 (13)	0.0005 (14)
C1	0.0258 (19)	0.030 (2)	0.0238 (19)	−0.0002 (16)	−0.0010 (15)	−0.0006 (16)
C2	0.024 (2)	0.049 (3)	0.029 (2)	0.0001 (19)	0.0010 (17)	−0.004 (2)
C3	0.044 (3)	0.035 (2)	0.030 (2)	−0.004 (2)	−0.0019 (19)	0.0095 (19)
C4	0.043 (3)	0.039 (3)	0.041 (3)	−0.005 (2)	−0.003 (2)	0.010 (2)
C5	0.062 (3)	0.044 (3)	0.048 (3)	−0.013 (3)	−0.018 (3)	0.015 (3)
C6	0.092 (5)	0.042 (3)	0.054 (4)	−0.012 (3)	−0.008 (3)	0.007 (3)
C7	0.120 (6)	0.052 (4)	0.046 (3)	0.007 (4)	0.012 (4)	−0.008 (3)
C8	0.074 (4)	0.049 (3)	0.042 (3)	0.001 (3)	0.003 (3)	−0.003 (3)
C9	0.057 (3)	0.054 (3)	0.060 (4)	0.002 (3)	0.013 (3)	0.004 (3)
C10	0.0262 (19)	0.031 (2)	0.028 (2)	−0.0005 (17)	0.0059 (16)	−0.0030 (17)
C11	0.025 (2)	0.041 (2)	0.030 (2)	−0.0028 (18)	0.0072 (17)	−0.0067 (19)
C12	0.037 (2)	0.038 (3)	0.041 (3)	−0.005 (2)	0.009 (2)	−0.010 (2)
C13	0.045 (3)	0.033 (2)	0.051 (3)	−0.004 (2)	0.012 (2)	0.001 (2)
C14	0.038 (2)	0.042 (3)	0.038 (3)	0.000 (2)	0.010 (2)	0.004 (2)
C15	0.038 (2)	0.037 (2)	0.027 (2)	−0.0035 (19)	0.0045 (18)	0.0002 (19)
C16	0.049 (3)	0.046 (3)	0.031 (2)	−0.009 (2)	0.010 (2)	−0.009 (2)

### Geometric parameters (Å, °)

**Table:**

Cl1—C2	1.771 (5)	C6—H6	0.9500
Cl2—C2	1.762 (4)	C7—C8	1.369 (8)
Cl3—C2	1.762 (4)	C7—H7	0.9500
P1—O2	1.468 (3)	C8—H8	0.9500
P1—N2	1.623 (4)	C9—H9A	0.9800
P1—N3	1.637 (3)	C9—H9B	0.9800
P1—N1	1.704 (3)	C9—H9C	0.9800
O1—C1	1.213 (4)	C10—C15	1.397 (6)
N1—C1	1.341 (5)	C10—C11	1.404 (5)
N1—H1	0.8800	C11—C12	1.390 (6)
N2—C3	1.439 (5)	C11—C16	1.499 (6)
N2—H2	0.8800	C12—C13	1.377 (6)
N3—C10	1.419 (5)	C12—H12	0.9500
N3—H3	0.8800	C13—C14	1.372 (6)
C1—C2	1.554 (6)	C13—H13	0.9500
C3—C4	1.371 (6)	C14—C15	1.373 (6)
C3—C8	1.420 (7)	C14—H14	0.9500
C4—C5	1.413 (7)	C15—H15	0.9500
C4—C9	1.472 (7)	C16—H16A	0.9800
C5—C6	1.369 (8)	C16—H16B	0.9800
C5—H5	0.9500	C16—H16C	0.9800
C6—C7	1.377 (9)		
			
O2—P1—N2	115.54 (18)	C8—C7—C6	119.1 (6)
O2—P1—N3	117.28 (17)	C8—C7—H7	120.4
N2—P1—N3	102.06 (17)	C6—C7—H7	120.4
O2—P1—N1	105.08 (15)	C7—C8—C3	120.0 (6)
N2—P1—N1	109.89 (18)	C7—C8—H8	120.0
N3—P1—N1	106.68 (16)	C3—C8—H8	120.0
C1—N1—P1	123.1 (3)	C4—C9—H9A	109.5
C1—N1—H1	118.4	C4—C9—H9B	109.5
P1—N1—H1	118.4	H9A—C9—H9B	109.5
C3—N2—P1	123.6 (3)	C4—C9—H9C	109.5
C3—N2—H2	118.2	H9A—C9—H9C	109.5
P1—N2—H2	118.2	H9B—C9—H9C	109.5
C10—N3—P1	127.1 (3)	C15—C10—C11	120.0 (4)
C10—N3—H3	116.4	C15—C10—N3	121.0 (4)
P1—N3—H3	116.4	C11—C10—N3	118.9 (4)
O1—C1—N1	125.2 (4)	C12—C11—C10	118.0 (4)
O1—C1—C2	120.1 (3)	C12—C11—C16	120.9 (4)
N1—C1—C2	114.7 (3)	C10—C11—C16	121.1 (4)
C1—C2—Cl2	110.2 (3)	C13—C12—C11	121.7 (4)
C1—C2—Cl3	107.5 (3)	C13—C12—H12	119.2
Cl2—C2—Cl3	110.0 (2)	C11—C12—H12	119.2
C1—C2—Cl1	110.1 (3)	C14—C13—C12	119.7 (4)
Cl2—C2—Cl1	108.8 (2)	C14—C13—H13	120.2
Cl3—C2—Cl1	110.3 (2)	C12—C13—H13	120.2
C4—C3—C8	120.6 (5)	C13—C14—C15	120.7 (4)
C4—C3—N2	121.2 (4)	C13—C14—H14	119.7
C8—C3—N2	118.1 (4)	C15—C14—H14	119.7
C3—C4—C5	118.4 (5)	C14—C15—C10	120.0 (4)
C3—C4—C9	121.0 (4)	C14—C15—H15	120.0
C5—C4—C9	120.5 (5)	C10—C15—H15	120.0
C6—C5—C4	119.9 (6)	C11—C16—H16A	109.5
C6—C5—H5	120.0	C11—C16—H16B	109.5
C4—C5—H5	120.0	H16A—C16—H16B	109.5
C5—C6—C7	121.9 (6)	C11—C16—H16C	109.5
C5—C6—H6	119.0	H16A—C16—H16C	109.5
C7—C6—H6	119.0	H16B—C16—H16C	109.5
			
O2—P1—N1—C1	176.0 (3)	N2—C3—C4—C9	−0.6 (7)
N2—P1—N1—C1	51.1 (4)	C3—C4—C5—C6	−0.6 (7)
N3—P1—N1—C1	−58.9 (4)	C9—C4—C5—C6	178.1 (5)
O2—P1—N2—C3	−29.9 (4)	C4—C5—C6—C7	1.6 (8)
N3—P1—N2—C3	−158.3 (4)	C5—C6—C7—C8	−1.2 (9)
N1—P1—N2—C3	88.8 (4)	C6—C7—C8—C3	−0.1 (8)
O2—P1—N3—C10	51.5 (4)	C4—C3—C8—C7	1.0 (8)
N2—P1—N3—C10	178.8 (3)	N2—C3—C8—C7	−177.7 (5)
N1—P1—N3—C10	−65.9 (4)	P1—N3—C10—C15	12.9 (6)
P1—N1—C1—O1	3.5 (6)	P1—N3—C10—C11	−165.8 (3)
P1—N1—C1—C2	−174.1 (3)	C15—C10—C11—C12	−0.3 (6)
O1—C1—C2—Cl2	3.5 (5)	N3—C10—C11—C12	178.4 (4)
N1—C1—C2—Cl2	−178.7 (3)	C15—C10—C11—C16	−179.1 (4)
O1—C1—C2—Cl3	−116.4 (4)	N3—C10—C11—C16	−0.3 (6)
N1—C1—C2—Cl3	61.4 (4)	C10—C11—C12—C13	0.7 (6)
O1—C1—C2—Cl1	123.5 (4)	C16—C11—C12—C13	179.4 (4)
N1—C1—C2—Cl1	−58.8 (4)	C11—C12—C13—C14	−0.1 (7)
P1—N2—C3—C4	93.0 (5)	C12—C13—C14—C15	−0.8 (7)
P1—N2—C3—C8	−88.3 (5)	C13—C14—C15—C10	1.2 (7)
C8—C3—C4—C5	−0.6 (7)	C11—C10—C15—C14	−0.6 (6)
N2—C3—C4—C5	178.1 (4)	N3—C10—C15—C14	−179.4 (4)
C8—C3—C4—C9	−179.3 (5)		

### Hydrogen-bond geometry (Å, °)

**Table:**

*D*—H···*A*	*D*—H	H···*A*	*D*···*A*	*D*—H···*A*
N1—H1···O2^i^	0.88	1.90	2.768 (4)	170.
N2—H2···O1^ii^	0.88	2.11	2.957 (4)	162.
N3—H3···O1^ii^	0.88	2.39	3.149 (4)	144.

Symmetry codes: (i) −*x*+1, *y*, −*z*+3/2; (ii) −*x*+1/2, −*y*+1/2, −*z*+1.
